# Evaluating protein complexes between human aquaporin and calmodulin using biomolecular fluorescence complementation

**DOI:** 10.1038/s41598-025-12865-z

**Published:** 2025-08-02

**Authors:** Jessica Glas, Johanna Landén, Kristina Hedfalk

**Affiliations:** Department of Chemistry and Molecular Biology, Gothenburg University, Göteborg, 405 30, Box 462 Sweden

**Keywords:** Aquaporin, Calmodulin, *Saccharomyces cerevisiae*, Bimolecular fluorescence complementation, Biochemistry, Molecular biology

## Abstract

Aquaporins (AQPs) are a family of integral membrane proteins crucial for the flow of water and other small molecules across cellular membranes. The involvement of calmodulin (CaM), a multifunctional calcium-binding protein, has emerged as a central regulator for specific aquaporin homologues from eukaryotes. Using a systematic approach, applying advanced high throughput screening methods *in vivo*, combining flow cytometry with microscopy, we have evaluated the putative interaction between CaM and the 13 human AQP homologues recombinantly produced in the yeast *Saccharomyces cerevisiae*. This comprehensive approach is complemented by a theoretical validation of potential CaM binding sites and a review of confirmed CaM binding locations from previous research. Our investigation is based on the established interaction of hAQP0 and CaM and we have successfully validated the binding of hAQP1 and hAQP4 to CaM. Noteworthy, discernibly high fluorescence frequency signals were observed for hAQP8 and hAQP9, which did not correlate with a particularly high production level, supporting protein complex formation with CaM for those AQP homologues. Overall, we present a systematic approach to screen novel membrane protein interactions *in vivo*, relying on co-expression in yeast of Bimolecular Fluorescence Complementation (BiFC) complexes providing new insights into the regulation of the hAQPs.

## Introduction

AQPs, a sub-family of integral membrane proteins, facilitate the passive flow of water across biological membranes while maintaining a transmembrane proton gradient^1^. In humans, there are 13 aquaporin homologues (hAQP0-hAQP12) expressed in various tissues such as brain, kidneys and lungs^2^. Some AQP isoforms also allow flow of neutral solutes, like glycerol, and flow of gases has also been reported^3^. They form tetramers, with each monomer containing a functional pore^4^ composed of six transmembrane (TM) α-helices (H1–H6), five connecting loops (loops LA–LE), and cytoplasmic N- and C-termini^5^. While sharing a common structural core, AQPs exhibit distinct structural variations in loops and termini, suggesting functional and/or regulatory roles in these domains^6^.

CaM, a ubiquitous eukaryotic protein with a molecular weight of approximately 17 kDa, serves as a primary intracellular receptor for calcium ions ([Ca^2+^]), playing a pivotal role in regulating a diverse array of cellular proteins^7^. It is evolutionarily conserved and consists of four EF-hand binding sites distributed between two globular domains, namely the N-lobe and the C-lobe, collectively binding four Ca^2+^ ions^8^. Upon Ca^2+^ binding, CaM undergoes conformational changes facilitating interaction with various target proteins^9^. Notably, CaM is often associated with membrane-spanning channels^10^ including aquaporins (AQPs)^11,12^.

Specifically, the complex formation between AQP0, the most abundant membrane protein in lens fiber cells^13^and CaM have been thoroughly studied by NMR, Electron Microscopy (EM) and biochemical analysis and the binding site is identified^14,15^. For AQP0, CaM binding has significant implications for water flow regulation where CaM influences the closing of AQP0 channels, modulating water flow across lens fiber membranes^16^. The binding occurs at a specific C-terminal stretch of AQP0 and is dependent on the presence of calcium ions^17^. CaM’s interaction thus inhibits the permeability of AQP0^18^, which is further demonstrated in experiments conducted on *Xenopus* oocytes^15^.

Similarly, CaM has been identified as a key regulatory player in AQP4^19^, highly expressed in the human brain and central nervous system (CNS)^20^. The interaction with CaM regulates AQP4’s subcellular localization, particularly at astrocyte endfeet, influencing bidirectional water flow across membranes. Abnormal loss of polarization in AQP4, observed in conditions like cytotoxic edema, is linked to CaM-mediated re-localization, emphasizing the potential significance of CaM inhibition in preventing such events and accelerating recovery from CNS edema^11^. A C-terminal binding site is identified in the AQP4 C-terminus^11^. However, Ishida et al. conducted a study that proposed an additional CaM binding site at the N-terminal region of AQP4^21^.

Furthermore, the study by Rabaud et al.^22^ proposes a potential CaM binding site at the N-terminus of AQP6, an anion channel primarily found in kidney cells. The interaction between AQP6 and CaM was observed by mixing AQP6 expressing CHO-K1 cells with CaM beads, in the presence of calcium. In addition, synthetic peptides mimicking the presumed CaM binding site of AQP6 along with a modified version of the N-terminal site, were assessed for their ability to bind to CaM. This analysis revealed a loss of CaM binding activity with the mutated N-terminal site.

Also, the interaction between AQP1 and CaM has been investigated in rats^23^. It was suggested that the rapid translocation of AQP1 depends not only PKC-mediated phosphorylation and calcium influx, but also on the activation of CaM. Its impact was shown using a CaM antagonist, which hindered the translocation of AQP1 in the astrocytes. Specifics regarding the precise binding location remain, however, elusive^23^.

Understanding the regulatory processes of aquaporins by Protein-Protein Interactions (PPIs) is crucial for exploring their involvement in diseases. Our investigation addresses the gap in understanding regarding the interaction between the whole repertoire of hAQPs and CaM. Such protein complexes have been reported in individual cases (hAQP0, hAQP1, hAQP4 and hAQP6) without a comprehensive analysis across the entire set of hAQPs. The current limitation is that previous experiments are performed using different *in vivo* approaches, commonly also relying on isolated protein or synthetic peptide studies. To provide a comparable analysis using the same system for all hAQP isoforms, we utilized the established Bimolecular Fluorescence Complementation (BiFC) screening method enabling validation of direct protein-protein interactions (PPIs) within tetrameric complexes initially established for hAQP1 and hAQP0 forming complexes to CaM. Through fusion of each target protein with a complementary YFP fragment, BiFC complexes were formed when expressed in *S. cerevisiae*^24^.

Subsequently, we optimized the screening procedure by employing population screening and quantification of BiFC complexes via flow cytometry, substantially enhancing throughput in our follow-up study^25^. Building upon this groundwork, we have now investigated the full set of hAQPs, incorporating appropriate control of non-forming complexes, while ensuring correct signal localization at the plasma membrane and considering individual expression levels of each of the hAQPs. This comprehensive and systematic approach aims to provide deeper insights into the broader impact of CaM on diverse hAQPs by analysing PPIs in living cells. Moreover, we supplement our experimental findings with insights from *in silico* analysis, comparing them with previous studies to identify potential binding sites in the AQP homologous.

## Results

### Theoretical prediction of CaM binding sites in the human AQPs

**Fig. 1:**
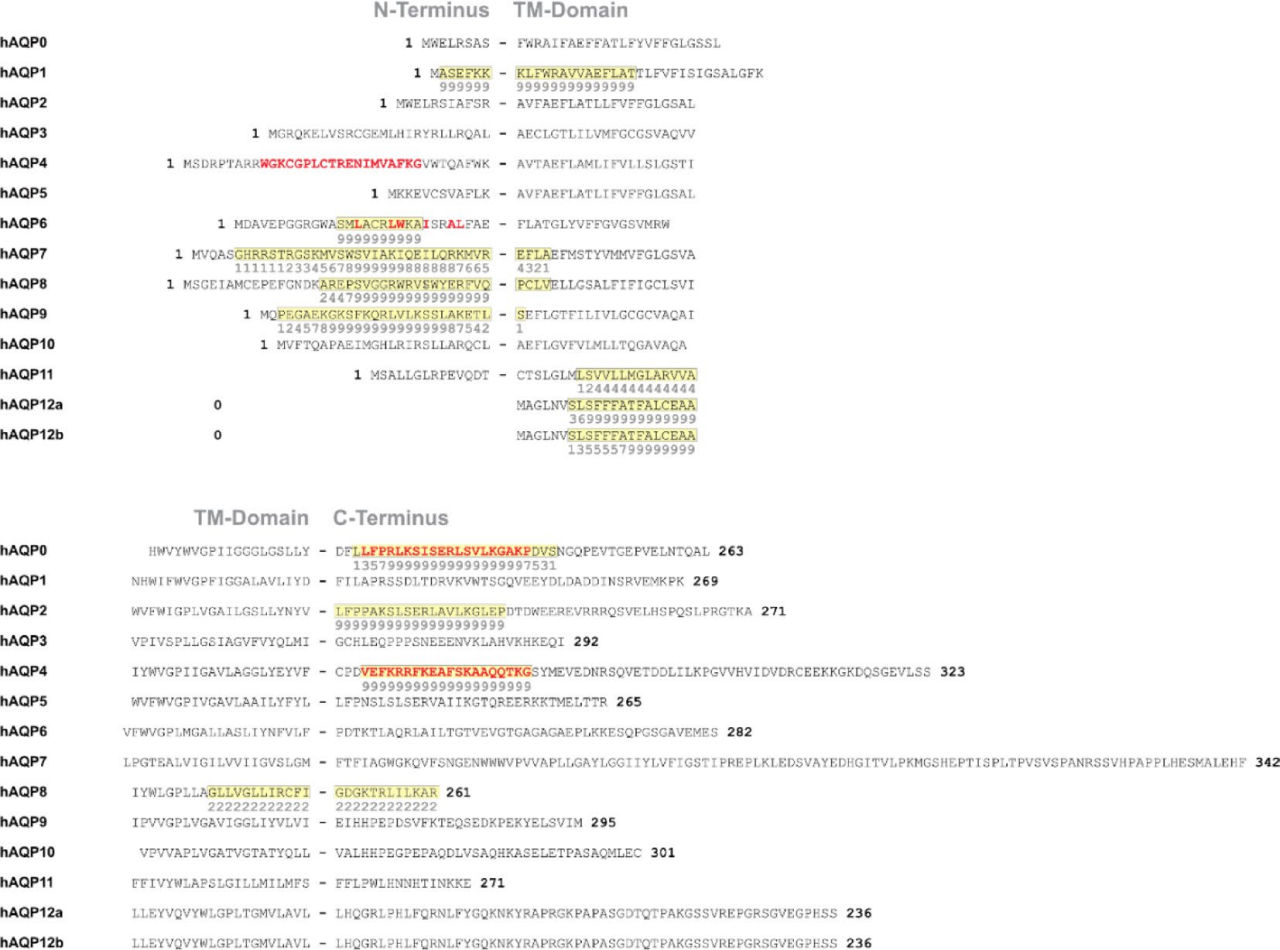
Theoretical CaM binding motifs in the N- and C-terminus of the human aquaporin homologues. The Calmodulin Target Database^26^ was used to identify potential CaM binding sites in hAQP0-hAQP12. The location of the putative CaM-binding motifs is highlighted in yellow and score 0–9 is used to indicate the strength of the binding. Additionally, interaction sites to CaM, which has been confirmed by experiments, are highlighted in red.

To explore putative hAQP-CaM protein complexes, we first examined the sequences of the hAQP homologues using the Calmodulin Target Database^26^ to determine scores for potential CaM binding sites in the N- and the C-terminus, respectively (Fig. 1) Some hits are identified in the TM domain of the proteins, which is considered negligible for our analysis. These hits are observed close to the N-terminus for hAQP1, hAQP11 and hAQP12a, indicated by high scores for hAQP1 and hAQP12a. In the C-terminal part of the hAQPs, there is only one example of a CaM binding site in the TM-domain: hAQP8 having a rather low score. Focusing on the hydrophilic extensions, strong CaM binding sites are identified in the N-terminus of five of the 13 hAQP homologues: hAQP1, hAQP6, hAQP7, hAQP8 and hAQP9. In the hydrophilic C-terminus, strong CaM binding sites are identified in three hAQP homologues, hAQP0, hAQP2 and hAQP4, along with the already mentioned weak CaM binding site suggested for hAQP8, stretching from the TM-domain towards the C-terminus. Noteworthy, hAQP3, hAQP5 and hAQP10 are not suggested to bind CaM in either terminus. Altogether, we conclude that the theoretical prediction of hAQP-CaM complexes confirms the established interactions between hAQP0, hAQP1, hAQP4 and hAQP6 with CaM, where the specific C-terminal binding sites have been experimentally confirmed for hAQP0 and hAQP4, respectively. Interestingly, complexes between CaM and hAQP7, hAQP8 and hAQP9 are also suggested, and especially for hAQP8 and hAQP9, involving strong binding as well as long stretches of the AQP N-terminal hydrophilic domains. In addition, there is a novel motif for CaM binding in the C-terminus of hAQP2. Furthermore, the proposed N-terminal binding site in hAQP6 is identified in the theoretical prediction while the one suggested in the hAQP4 N-terminus is not recognized.

### Quantification of hAQP-CaM BiFC complexes using flow cytometry

To experimentally evaluate putative hAQP-CaM interactions, we employed our previously established BiFC and Flow Cytometry assay, enabling *in vivo* validation of membrane protein complexes in a high-throughput manner^25^. For this purpose, hAQPs were cloned into plasmids, incorporating the YFP_N_ tag at the N-terminus of the aquaporins (Fig. S1). These plasmids were co-transformed into *S. cerevisiae* alongside a second plasmid containing the YFP_C_-CaM construct. Analysis adhered to the protocol outlined in our prior published study, suggesting this cloning strategy for hAQP-CaM complexes. As a control for a non-constructive complex, we concomitantly examined the interaction between CaM and AQP0ΔC, a variant of AQP0 with a stop codon at amino acid position 221 abolishing the CaM interaction site in the C-terminus^15^.

**Fig. 2:**
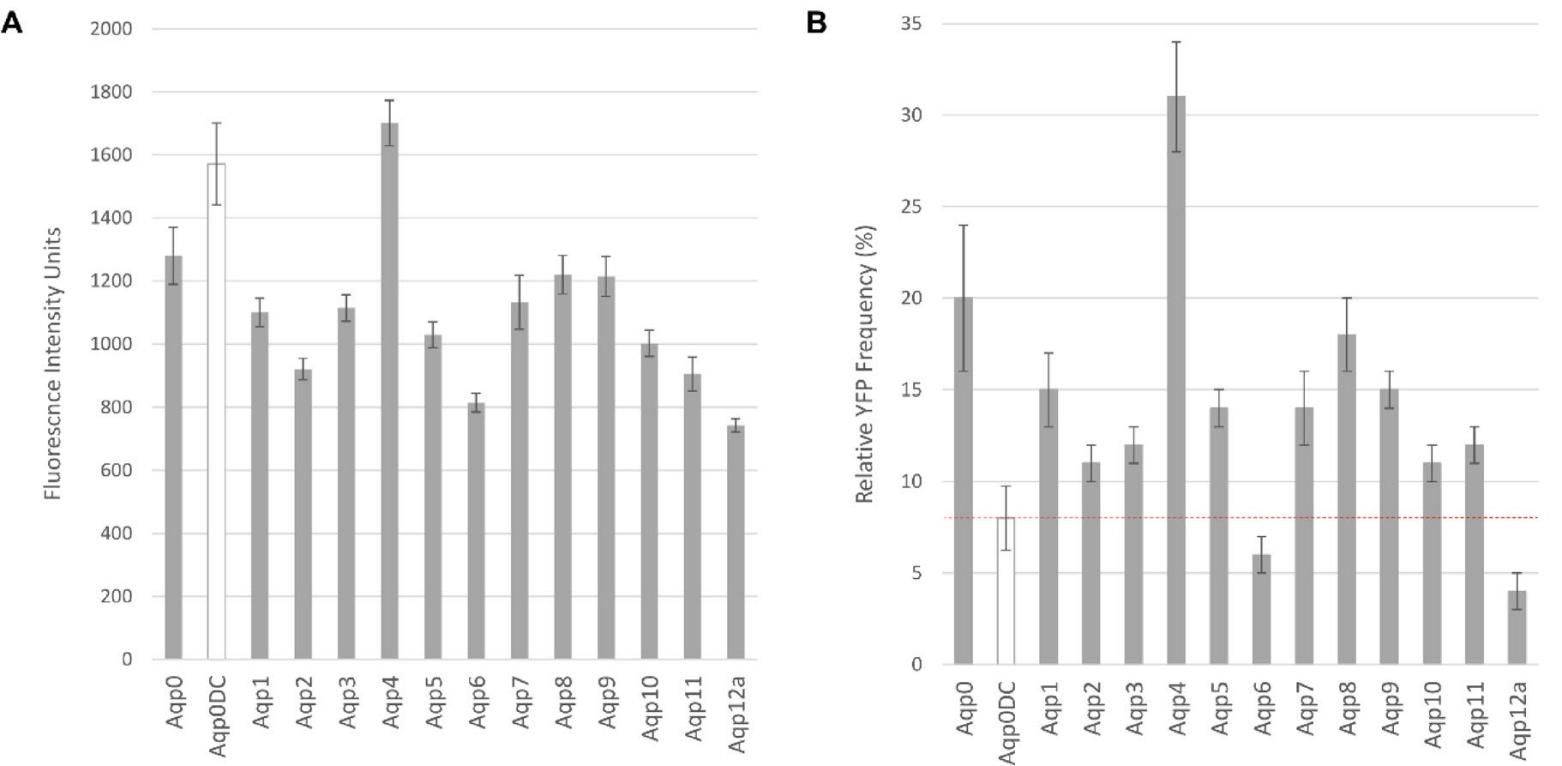
Quantification of BiFC signal through flow cytometry from yeast cells producing complexes of CaM and each of the hAQP homologues (**A**) The average fluorescence intensity and standard error of mean are displayed for each hAQP-CaM pair. (**B**) The average percentage of fluorescent cells and standard error of the mean are shown for each hAQP-CaM pair.

For each pair of constructs, a thorough setup of biological and technical repeats was applied, involving three independent transformation events, each with ten technical replicates. Fluorescence intensity as well as frequency data were assessed from a minimum of 10,000 events per measurement to ensure a valid representation of the entire population (Fig. 2, Table S1).

*S. cerevisiae* cells co-transformed with the plasmids encoding hAQP0 and CaM, as well as hAQP4 and CaM, exhibited the highest average fluorescence intensity (1280 +/- 130 and 1700 +/- 72), Fig. 2A, and frequency (20% +/- 4 and 31% +/-3), Fig. 2B, which correlates well with confirmed protein complexes with CaM for these two AQP homologues. As already described in^25^the main criterium for validating real protein complex formation in this assay is not the fluorescence intensity as any complementation of the YFP molecule leads to a similar fluorescence signal. Nonetheless, such events are notably less frequent in proteins lacking interaction, highlighting the fluorescence frequency signal as a more reliable evaluation for detecting constructive complex formation. Thus, deleting the C-terminus of hAQP0 (223–242) resulted in a significant decrease in fluorescence frequency, aligning with earlier research^24,25^. hAQP6 and hAQP12 showed the lowest fluorescence frequency signals at 3% and 6%, respectively which was even lower than our negative control AQP0ΔC with CaM (8%). On the contrary, high fluorescent signals are observed for hAQP1, along with hAQP8 and hAQP9 suggesting constructive complex formation of these AQP homologues with CaM. In comparison, hAQP2, hAQP3, hAQP5, hAQP7, hAQP10 and hAQP11, showed an intermediate frequency, ranging from 11 to 14%. Noteworthy, statistical analysis revealed a significant different Relative YFP Frequency signal to the AQP0ΔC negative control of all hAQPs (Table S1).

### Verification and localization of hAQP-CaM BiFC complexes in yeast cells

**Fig. 3:**
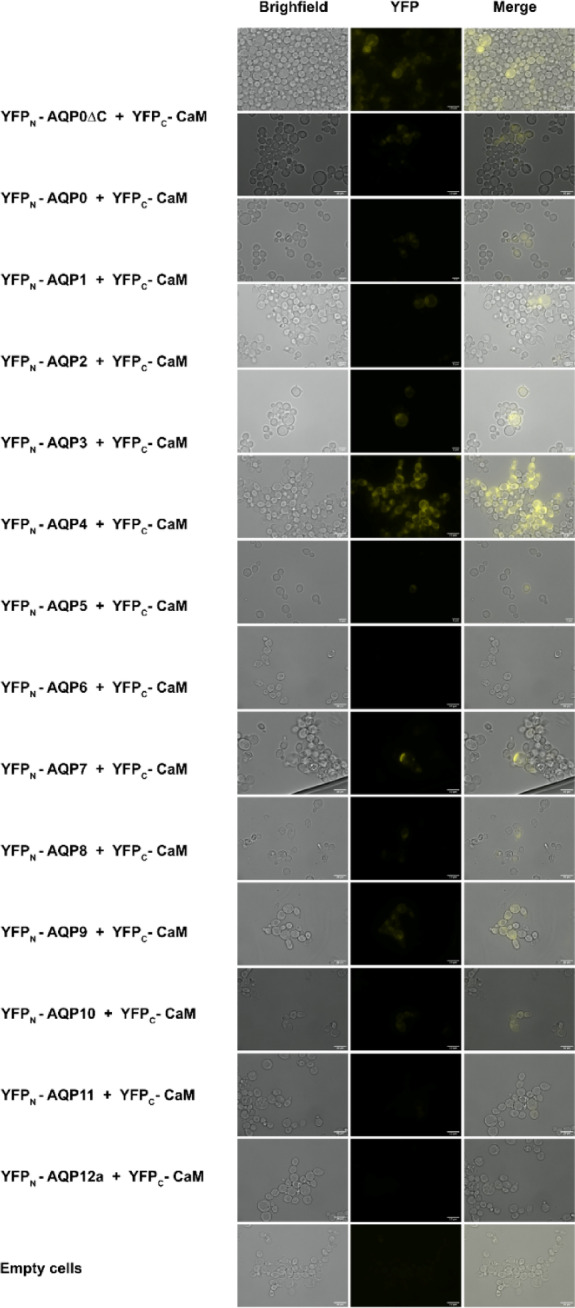
Fluorescence and bright-field images of BiFC hAQP-CaM complexes produced in *S. cerevisiae* cells and images where both channels were merged. The strongest fluorescence signal was achieved from YFP_N_-AQP4 + YFP_C_-CaM, clearly indicating a constructive complex formation, as well as from YFP_N_-AQP0 + YFP_C_-CaM. Weak fluorescence was observed for the BiFC complexes showing low to intermediate fluorescence frequency. No fluorescence was observed in cells producing YFP_N_-AQP6 + YFP_C_-CaM, YFP_N_-AQP11 + YFP_C_-CaM, YFP_N_-AQP12 + YFP_C_-CaM as well as untransformed *S. cerevisiae* cells, providing a better negative control for the microscopy analysis than the BiFC formation control lacking the AQPO C-terminus, YFP_N_-AQP0ΔC + YFP_C−_CaM.

To verify that the fluorescent signal is related to proteins properly produced in yeast, we also evaluated the recombinant yeast cells by microscopy (Fig. 3). In untransformed *S. cerevisiae* cells, there is no observable fluorescence as no YFP fragment is transformed into the cells, providing a proper negative control for the qualitative evaluation. Similarly, *S. cerevisiae* cells transformed with the combinations YFP_N_-AQP6 + YFP_C_-CaM and YFP_N_-AQP12 + YFP_C_-CaM exhibited no detectable fluorescence, an observation in good agreement with low fluorescence frequency when analyzed by flow cytometry. The most robust fluorescence signal was observed for the YFP_N_-AQP4 and YFP_C_-CaM combination, where a significant portion of cells displayed this fluorescence signal. In cell fluorescence is also observed for the other hAQPs showing constructive BiFC complexes (Fig. 2, Table S2): hAQP0, hAQP1, hAQP8 and hAQP9. Interestingly, fluorescent signals are also observed for cells producing hAQP-CaM complexes showing intermediate fluorescent frequency (hAQP2, hAQP3, hAQP5, hAQP7 and hAQP10), while no fluorescence is observed for hAQP11, illustrating that fluorescence microscopy is a poor tool for quantitation and better suited for qualitative evaluation. Furthermore, the non-interaction control, YFP_N_-AQP0ΔC + YFP_C_-CaM, a few cells exhibited a fluorescent signal which correlates with the high fluorescence intensity observed for this specific combination. In conclusion, the complementing microscopy analysis provides a qualitative analysis tool where only major differences can be evaluated together with some insight into the cellular localization of the fluorescent signal.

### Evaluation of the hAQP levels in the membrane of *S. cerevisiae*

Even though it is crucial to acknowledge the inherent biological variations associated with using a single cell for testing production level in the whole cell, verification of aquaporin production levels in the membrane fraction represents a critical step in confirming that the fluorescent signal originates from a properly localized protein.

To validate the production of all hAQPs and assess the production levels within the yeast cell membrane, we conducted Immunoblot assay utilizing an antibody specific to the YFP_N_ segment, as previously employed in our earlier study^25^. Notably, we enhanced the sample preparation process by conducting small-scale membrane preparations of individual colonies regrown to an optical density (OD_600_) of 0.5, aiming to harvest the samples at the same stage of the logarithmic growth, and we also standardized the protein quantity in every sample to assist accurate comparison. A negative control, consisting of untransformed *S. cerevisiae* cells (Empty cells), was included to discern nonspecific bands from those specifically associated with hAQPs. The expression of YFP_C_-CaM in *S. cerevisiae* cells has already been confirmed in our previous study and is therefore not a subject of investigation^25^.

**Fig. 4:**
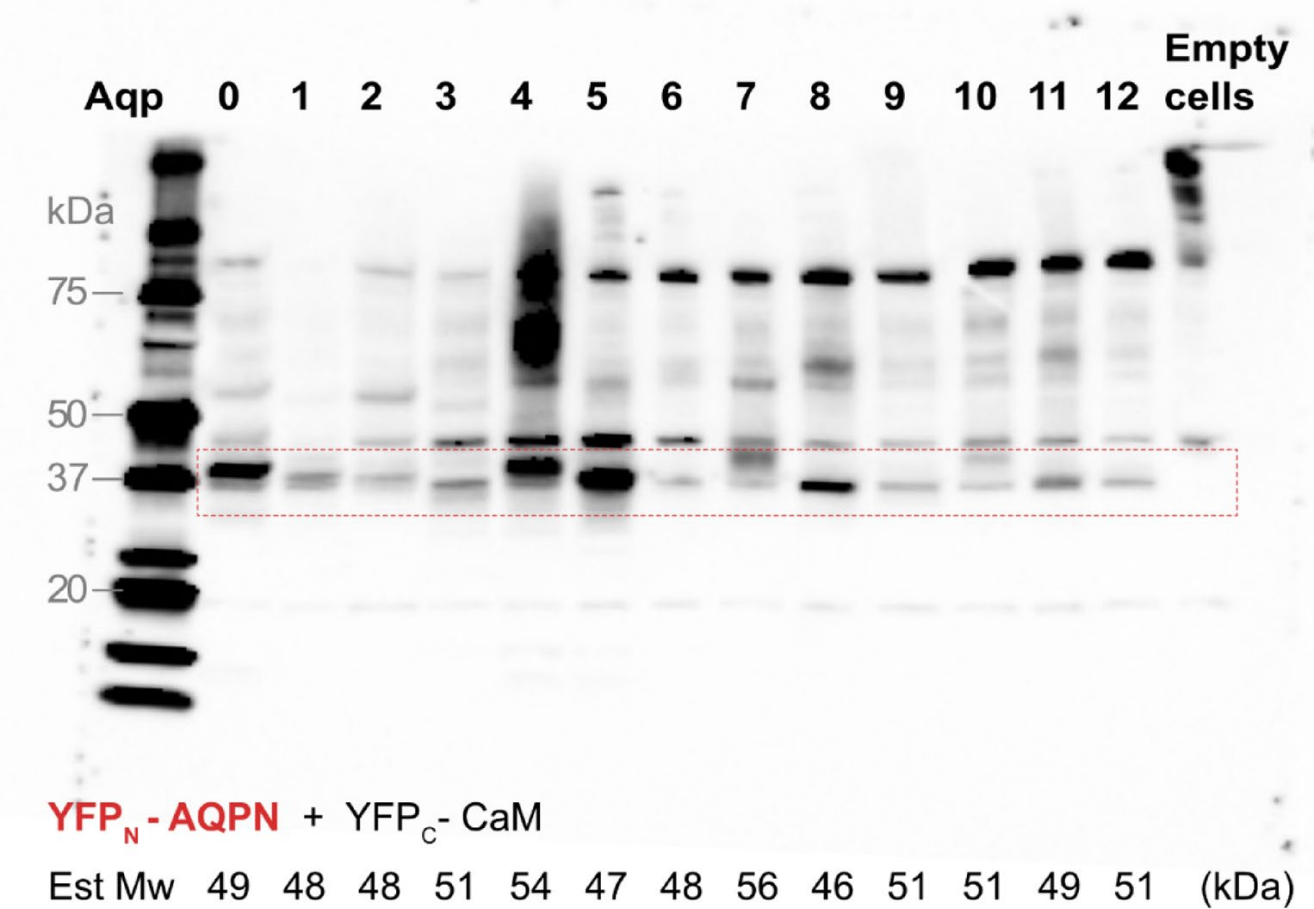
Immunoblot analysis of the different hAQP-CaM BiFC complexes. To analyze and compare different production levels of the hAQP-CaM complexes, Immunoblot using a primary antibody towards the YFP_N_-hAQP fragment was applied. Total protein, 30 µg, was loaded for each sample and even loading was confirmed by Ponceau staining of the membrane before incubation with the primary and secondary antibody, respectively. A typical Immunoblot is shown, highlighting the area of interest, and the estimated fragment size (Mw) is listed below for each hAQP-CaM complex. As is commonly observed for membrane proteins, the YFP_N_-hAQP fragments migrate at a slightly lower molecular weight than the one predicted theoretically.

While this analysis indicated constructive BiFC complexes at approximately 75 kDa, the predominant signals on the Immunoblot corresponded to the YFP_N_-AQP fragments, indicating an abundance of expressed fragments relative to the formation of BiFC complexes after treatment with Sodium Dodecyl Sulfate (SDS) (Fig. 4). Immunoblot signals are observed for all hAQP homologous, and consistent with previous observations^25^specific bands were noted to migrate at a lower molecular weight than their expected sizes (46–54 kDa). Upon comparing the production levels of different human aquaporins, notable distinctions emerge. The lowest production level is observed for hAQP2, hAQP6, hAQP7, hAQP9, hAQP10 and hAQP12a. All of those, except hAQP9, yield a correspondingly low or intermediate fluorescence frequency signal in the assay as well as in the microscopy evaluation of whole yeast cells, not supporting complex formation with CaM for those AQPs. In comparison, AQP9 being the exception, has high fluorescence frequency combined with low production levels, indeed supporting constructive complex formation with CaM. hAQP1 is produced to rather low levels in the membrane, but has a high fluorescence frequency, supporting a constructive BiFC complex formation with CaM. Conversely, hAQP0, hAQP4, hAQP5, and hAQP8 demonstrate the highest expression levels. Especially for hAQP0 and hAQP4, we see a clear correlation between a high production level and a high fluorescence frequency, as expected for established hAQP-CaM complexes. Noteworthy, hAQP8 shows similarly high fluorescence frequency combined with high production level, comparable to hAQP0, indicating a constructive complex formation with CaM. Interestingly, for both hAQP8 and hAQP9, strong CaM binding motives are identified in the N-terminus of both these hAQP homologues (Fig. 1), further supporting constructive complex formation (Table S2).

## Discussion

### Established hAQP-CaM complexes are confirmed by our comprehensive analysis

Our study employed a robust assay to evaluate interactions between hAQPs and CaM, providing insights into their binding dynamics. Unlike previous investigations, that focus only on selected aquaporins using a great variation of methods, we analyze direct interaction of full-length aquaporin proteins in real time in an *in vivo* assay. With multiple biological and technical replicates for each tested complex and careful consideration of varying production levels, our approach provides a comprehensive understanding of these interactions. Commonly, we observed a strong correlation between aquaporin expression levels and the YFP fluorescence frequency signals, which generally corresponded with detectable YFP signals in microscopy studies. In Table S2, we provide a comprehensive overview of our findings regarding the interaction between CaM and hAQPs, incorporating flow cytometry data, microscopy observations, expression levels, together with the theoretical predictions for binding sites and information on those that are experimentally confirmed (AQP0, AQP4 and AQP6).

Among the constructive BiFC pairs, YFP_N_-hAQP4 and YFP_C_-CaM exhibited the strongest interaction signal, characterized by the highest YFP intensity and frequency signals and the peak expression level of hAQP4. Notably, this interaction complex displayed pronounced strength compared to others, possibly due to dual interaction sites, one in each terminus (Fig. 1). This is supported by a recent study that proposes a second interaction site for hAQP4 at the N-terminus^21^although this site did not align with our *in silico* analysis findings. The BiFC complex involving YFP_N_-hAQP0 and YFP_C_-CaM, which showed the second-highest YFP frequency signal, also exhibited substantial expression levels, suggesting an interaction. These findings are strongly corroborated by prior research, which has identified the binding site for CaM in hAQP0, located at residues 223–242^15^, and for hAQP4 within residue 256–275^11^, which also aligns with the prediction of strong binding motifs using the Calmodulin Target Database^26^.

Furthermore, our assay not only confirmed the presence of a C-terminal binding site for CaM in hAQP0, consistent with previous studies^14,15^it was further confirmed by a construct lacking that specific sequence (YFP_N_-hAQP0ΔC + YFP_C_-CaM). Interestingly, deletion of the C-terminus reduced YFP frequency while maintaining high fluorescence intensity, supporting that frequency may be a primary indicator of real complex formation. We hypothesize that fluorescence intensity reflects the duration of the complex formation process and that the strength of the YFP signal might depend on the time when the complex was formed and how fluorescence diminishes over time. This observation is further supported by microscopy images showing a significant proportion of fluorescent cells. However, the higher proportion of fluorescent cells in this case, compared to other samples, is not interpreted as evidence of interaction but rather serves as a qualitative tool to confirm signal localization accurately.

In our previous investigation^24^we employed the BiFC assay to validate the interactions between hAQP1 and CaM, assessing YFP signal presence via fluorescence microscopy. Acknowledging the limitations inherent to this approach, we reevaluated the hAQP1 and CaM interaction using flow cytometry, factoring in protein production levels. Despite observing a relatively strong fluorescence signal in the hAQP1 and CaM complex, the concurrent low production level suggests a potential for complex formation.

In a prior study focusing on synthetic peptides^22^the interaction between hAQP6 and CaM highlighted the significance of specific amino acids within the N-terminus for CaM binding activity. Notably, three of these amino acids corresponded to residues within the predicted binding site (Fig. 1). However, our study observed a notably low fluorescence frequency signal for the hAQP6-CaM complex, likely due to the inherently low production level of hAQP6 in our assay system. Challenges in producing hAQP6 in yeast cells have been documented previously. For instance, attempts to overproduce hAQP6 in *P. pastoris* yielded very low yields, as noted in prior investigations^27^. The difficulties in achieving robust expression levels of hAQP6 may hinder the verification of its interaction with CaM using our assay. Similarly, the low fluorescence frequency observed for hAQP12, coupled with the diminished Immunoblot signal, may be attributed to the poor integration of certain hAQP isoforms into the yeast cell membrane, as previously observed^28^.

### First indication of complex formation between hAQP8 and hAQP9 with CaM

Interestingly, the combinations YFP_N_-AQP8 + YFP_C_-CaM and YFP_N_-AQP9 + YFP_C_-CaM manifest remarkably similar high YFP frequency signals at 18% and 15%, respectively, in a similar range as YFP_N_-AQP1 + YFP_C_-CaM (Fig. 2). Notably, YFP_N_-AQP8 exhibits significantly stronger expression than YFP_N_-AQP9. This disparity in expression levels prompts insights into the nature of interactions. Despite hAQP9’s lower expression level, the comparable YFP frequency and higher YFP signal in the microscopy study suggest a potentially stronger interaction than is displayed in the flow cytometry data and might even be stronger between hAQP9 and CaM than between hAQP8 and CaM. In conclusion, our study indeed indicates that the hAQP8 and hAQP9 isoforms form protein complexes with CaM, which to our knowledge is shown for the first time. However, even though strong theoretical binding motifs for CaM are predicted in the N-terminus of both hAQP8 and hAQP9 (Fig. 1), the precise binding site of CaM (N- or C-terminus) remains experimentally undetermined. Despite their similar overall structure, the hydrophilic extension of the proteins lack conservation among aquaporin family members, suggesting potential targets for different regulatory mechanisms. To determine the exact binding site for CaM of hAQP1, hAQP4, hAQP8 and hAQP9, it would be essential to generate and test mutants deleting both the N- and C-termini.

hAQP8 and hAQP9 belongs to the AQP homologous that are least investigated and there are no high-resolution structures of those, as compared to bAQP0^29^, hAQP1 ^4^, and hAQP4^30^, for which there are confirmed AQP-CaM complexes for AQP0 and AQP4 (Table S2). Both hAQP8 and hAQP9 facilitate the water flow of hepatic bile^31^. Further, hAQP8 is localized to the midpiece of the sperm where its main function is elimination of hydrogen peroxide, thereby being important for the response to oxidative stress as well as normal fertility^32^. hAQP9 is the dominating water channel in the liver, facilitating flow of glycerol and hydrogen peroxide, having several implications for diseases as well as the potential use as biomarker^33^. Noteworthy, hAQP9 is suggested to play a role in septic shock, hence being a putative target for novel therapeutics^34^. In contrast to hAQP0 and hAQP4, where the interaction with CaM has been thoroughly studied, very little is known about the putative complexes between hAQP1 and hAQP6 with CaM^35^. In addition, a novel interaction between hAQP8 and hAQP9 with CaM, as suggested by this study, could therefore have the potential to contribute to the multifaceted map describing the molecular mechanisms of crucial membrane protein targets in specific cells and tissues and future studies in more complex cell types are therefore of high relevance.

### Production levels and cloning strategies are important parameters

Although we observed relatively high YFP frequency signals for some aquaporins where we didn’t predict an interaction with CaM, we attribute this to their relatively high production levels, which lead to elevated YFP background signals. For instance, hAQP5 demonstrates production levels comparable to hAQP4. However, despite this similarity in production levels, the fluorescence frequency level associated with hAQP5 is significantly lower than that observed with hAQP4 (14% vs. 31%). This discrepancy suggests a lack of real complex formation between CaM and hAQP5. This observation aligns with predictions from our *in silico* analysis, which did not identify potential interaction sites between hAQP5 and CaM. The relatively high fluorescence frequency level observed in hAQP5 may be attributed to its elevated production level, resulting in an increased fluorescence background signal. This phenomenon highlights the importance of distinguishing between true interaction signals and background noise when assessing protein-protein interactions.

Another factor to consider is the putative interaction between recombinant aquaporins and endogenous CaM in *S. cerevisiae*, expressed from the CMD1 gene^36^. Notably, in yeast, CaM is required for endocytosis and it has a key role in stress-activated signaling pathways^37^. Intuitively, a possible influence from endogenous CaM on the BiFC Aqp-CaM complex cannot be excluded. Hence, when verifying complex formation, preferably using mammalian cells, membrane localization should be addressed more carefully. Ideally, pure protein should be used for detailed characterization, which should be possible for most aquaporin isoforms, based on their reliable production in the related yeast species *P. pastoris*^27^.

In addition to aspects raised above on the differences in production levels of hAQPs and putative interaction with endogenous CaM, limitations of the assay arise from the selected cloning strategy, which may impact the complementation of the YFP molecule. The decision to attach the YFP molecule N-terminally to the hAQPs protein introduces the potential for steric hindrance^38^. This could impede the YFP molecule’s ability to reconstitute its three-dimensional structure, especially when the interaction site is presumed to be at the N-terminus of the AQP protein. Future studies could explore alternative cloning strategies, such as attaching YFP molecules to the C-terminus, to enhance complex formation probability. Moreover, our cloning strategy significantly influences the results. Previous investigations have shown that selecting a longer linker sequence between the YFP fragment and the target protein results in a higher average fluorescence frequency value, suggesting a higher likelihood of YFP fragments maturing into a complex. This finding underscores the importance of considering the impact of the cloning strategy on assay outcomes and suggests avenues for optimization to improve experimental reliability and accuracy.

## Conclusions

In conclusion, our study offers an accurate examination of the potential interaction between calmodulin and the 13 aquaporin isoforms in human, shedding light on possible regulatory complexes.

Notably, our results confirm the established regulatory complexes between hAQP0, hAQP1 and hAQP4 to CaM, also coinciding with high production of these AQP isoforms in the yeast system. Additionally, we present indications of potential interactions between hAQP8 and hAQP9-CaM pairs, shedding new light on previously unexplored interactions. In essence, our comprehensive approach not only validates known interactions but also uncovers novel insights into the complex interplay between calmodulin and human aquaporins, contributing to important understandings into the regulatory mechanisms of these membrane proteins.

## Materials and methods

### Gene sequences

The study did not involve direct participation of human subjects. The CaM gene was graciously provided by Rachel Klevit from the University of Washington, while the human AQP0 gene, optimized for expression in yeast, was procured from Genscript (Piscataway, NJ). Additionally, Karin Lindkvist, Lund University, contributed with the plasmid containing full-length hAQP7. For the production of the hAQPs in *Pichia pastoris* described previously, silent mutations were introduced around the start methionine to resemble the yeast consensus sequence AAA ATG TCT^28^. The same approach was applied for the production in *S cerevisiae*, and the attB cloning site for the Gateway cloning was additionally added to the PCR primer. The transformation process involved the yeast strain *S. cerevisiae* (MYA-1662™ his3, ura3-52).

### Cloning of the constructs using Gateway^®^ technology

Gateway cloning vectors from Addgene specifically designed for high-throughput analyses in *S. cerevisiae*^39^ were modified by replacing the eGFP gene with either the YFP_C_ or YFP_N_ part as following the BiFC approach^24^where YFP_C_ or YFP_N_ consisted of amino acids 1 to 154 and 155 to 239 of the SYFP2 gene, respectively. This modification involved traditional restriction enzyme cloning, wherein the initial Gateway vectors intended for N-terminal tagging were subjected to digestion using *Spe*I and *Mab*I enzymes. The YFP_C_ or YFP_N_ genes were subsequently amplified through PCR and integrated into the cleaved vectors via ligation into the specified restriction sites.

Entry Clones for the study were prepared using attB PCR Primers for the amplification of the hAQP isoforms and CaM, respectively, and each PCR product was cloned into the donor vector pDONR222 with BP clonase. Since the cloned gene sequences were created by PCR, the resulting plasmids were verified by Sanger Sequencing to confirm that no mutations were accidentally introduced. Verified sequences were then cloned into the created Gateway vectors, containing the YFP fragments (YFP_C_ or YFP_N_, respectively) using LR clonase. The YFP fragment was fused N-terminally to the protein of interest, with a flexible linker sequence between the two proteins (GGPGGGH*QTSLYKKAGF*, where the italic stretch of amino acids is introduced by the attB cloning site). Hence, the resulting donor vectors, YFP_N_-hAQP0-12 and YFP_C_-CaM, respectively, included YFP_N_/YFP_C_ – linker -- hAQP0-12/CaM (Fig. S1).

### Chemical transformation of *S. cerevisiae* and fluorescence analysis using flow cytometry

For chemical transformation of BiFC construct into *S. cerevisiae cells* we followed the protocol previously described^25^. In short, cells with a HIS/URA deficiency were grown overnight at 30 °C in YPD medium and regrown the next day from a starting OD_600_ of 0.25 to an OD_600_ between 0.7 and 1.0. Cells were washed in H_2_0 followed by 100 mM LiAc and then fractionated into 50 µL aliquots, with each aliquot mixed with transformation mix (240 µL PEG4000 (50%), 36 µL 1 M LiAc, 50 µg denatured salmon sperm and 1 µg plasmid DNA). Cells were incubated at 30 °C for 30 min and then heat shocked at 42 °C for 25 min. After briefly spinning the cells, they were plated out onto -HIS -URA selective Media (SC) plates and incubated at 30 °C for at least 3 days. At least 10 individual colonies were randomly selected and regrown on SC agar plates for one night at 30 °C. The next day, colonies were inoculated in 5 mL SC medium at 30 °C with shaking overnight. The next day, cells were diluted to OD_600_ = 0.2 and regrown in 12-well tissue culture plates to OD_600_ = 0.5. For fluorescence measurements of BiFC complexes, transformed cells were evaluated using FACSMelody (BD) flow cytometer (100 μm nozzle size, blue excitation laser at 488 nm). 500 µL of a well resuspended sample was loaded into the FACS instrument. Fluorescence intensity of 100.000 cells of the live population was measured by excitation at 488 nm and evaluation in the FITC channel (527/32 nm bandpass filter). Debris and discordant cells were excluded by gating on FSC-A vs. SSC-A to identify yeast cells by size. Fluorescence frequency and intensity of gated cells were used for evaluation.

### Immunoblot analysis

To verify equal expression of all BiFC constructs, overnight liquid cultures of transformed cells were grown in 100 mL SC Medium to an OD_600_ = 0.5. Cells were harvested by centrifugation and resuspended in lysis buffer (20 mM Tris, pH 7.6; 100 mM NaCl; 0.5 mM EDTA and 5% Glycerol) and Fast Prep was used to open the cells using glass beads (speed 6.5, 5x, 45 s). The supernatant was spun down at 500xg, for 10 min at 4 °C resulting in a weakly yellow supernatant which was spun down at 10 000xg, 30 min, 4 °C followed by an additional centrifugations step of the supernatant (100 000xg, 90 min, 4 °C) to collect the membrane fraction. The resulting membrane pellet was resuspended in 100 µL lysis buffer and for the protein concentration was determination using the BCA assay. For each sample, 30 µg of the total protein content was used for Immunoblot analysis. The protocol described in^25^ was followed, except that the membranes were blocked in 3% milk powder solution for 1 h and the first antibody (anti YFPN: BioLegend #902601) was incubated overnight.

### Fluorescence microscopy

For the fluorescence microscopy images we followed the protocol in^25^ except that we resuspended the re-grown cultures in 10 µL H_2_0.

### Statistical analysis

An unpaired two component t-test with Welch’s correction was used to determine statistical difference between the control groups (*p* < 0.0001).

### Prediction of binding sites

We utilized the Calmodulin Target Database from the University of Toronto^26^ to predict the presence of calmodulin-binding motifs in aquaporins. Theoretical predictions obtained from the database’s “binding site search” yielded multiple hits. Our analysis focused on the N- and C-terminal sequences of the aquaporins for comprehensive assessment.

## Supplementary Information

Below is the link to the electronic supplementary material.

Supplementary Material 1

## Data Availability

Please contact the corresponding author (kristina.hedfalk@gu.se) if you want to request data from this study.
