# Supplementary material for: Evaluating protein complexes between human aquaporin and calmodulin using biomolecular fluorescence complementation
Source: Sci Rep. 2025 Aug 2;15:28203. doi: 10.1038/s41598-025-12865-z (PMC12317118; doi:10.1038/s41598-025-12865-z)
Supplement: Supplementary file 1 — Supplementary Material 1 [file 41598_2025_12865_MOESM1_ESM.pdf]

Supplementary Material

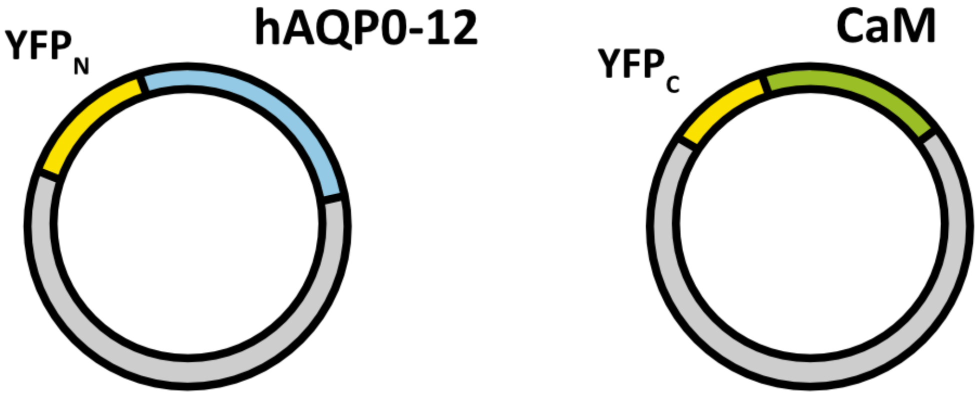

Figure S1. BiFC constructs for evaluation of aquaporin-CaM complexes, where the N-terminal YFP fragment is fused to the N-terminus of the aquaporin (YFP_N-hAQP0-12) and the C-terminal YFP fragment is fused to the N-terminus of CaM (YFP_C-CaM).

Table S1. Experimental setup for the evaluation of the fluorescence intensity and relative YFP frequency of BiFC complexes, specifying the number of biological repeats (independent transformation event) and technical repeats are shown for each construct. Each measurement involved a minimum of 10.000 cells. An unpaired two component t-test with Welch's correction was used to determine statistical difference of the Relative YFP Frequency, as comparted to the YFP$_N$-AQP0ΔC + YFP$_C$-CaM value. High fluorescence frequency is highlighted in green, intermediate in yellow and low in red.

YFP$_N$- AQPN + YFP$_C$- CaM	Biological Repeats n	Technical Repeats n	Fluorescence Intensity Units average	Fluorescence Intensity Units SEM	Relative YFP Frequencey average %	Relative YFP Frequencey SEM	Welch's unpaired t-test	Significance
**Aqp0**	3	30	1280	90	20	4	<0.0001	Yes
**Aqp0ΔC**	3	32	1571	130	8	1,75	-	-
**Aqp1**	3	30	1100	45	15	2	<0.0001	Yes
**Aqp2**	3	30	920	34	11	1	<0.0001	Yes
**Aqp3**	3	30	1114	42	12	1	<0.0001	Yes
**Aqp4**	3	30	1701	72	31	3	<0.0001	Yes
**Aqp5**	3	30	1029	41	14	1	<0.0001	Yes
**Aqp6**	3	30	814	29	6	1	<0.0001	Yes
**Aqp7**	3	30	1132	86	14	2	<0.0001	Yes
**Aqp8**	3	30	1220	61	18	2	<0.0001	Yes
**Aqp9**	3	30	1214	63	15	1	<0.0001	Yes
**Aqp10**	3	30	1002	42	11	1	<0.0001	Yes
**Aqp11**	3	30	905	54	12	1	<0.0001	Yes
**Aqp12a**	3	30	743	21	4	1	<0.0001	Yes

Table S2. Summary of the investigation of the interaction between CaM and hAQPs, including theoretical predictions, flow cytometry analysis, microscopy signal and expression levels verified by Immunoblot analysis: ++++ = very strong signal; +++ strong signal; ++ = signal; + = very low signal. Confirmation of established hAQP-CaM complexes are shown in green, not confirmed in red, and novel complexes in blue.

Aqp	Binding site for CaM Experimental evaluation	Binding site for CaM Theoretical evaluation	Fluorescence Intensity	Fluorescence Frequency	Microscopy	Immunoblot
**Aqp0**	C-terminal	C-terminal	++	++	+	+++
**Aqp0ΔC**	-	-	+++	-	+	
**Aqp1**	-	N-terminal	+	+	+	++
**Aqp2**	-	C-terminal	-	+	+	+
**Aqp3**	-	-	+	+	+	++
**Aqp4**	N, C-terminal	C-terminal	+++	+++	+++	++++
**Aqp5**	-	-	+	+	+	++++
**Aqp6**	N-terminal	N-terminal	-	-	-	+
**Aqp7**	-	N-terminal	+	+	+	+
**Aqp8**	-	N-terminal	++	++	+	+++
**Aqp9**	-	N-terminal	++	++	++	+
**Aqp10**	-	-	+	+	+	+
**Aqp11**	-	-	+	-	-	++
**Aqp12a**	-	-	-	-	-	+
